# Clinical Decision Support System for Guidelines-Based Treatment of Gonococcal Infections, Screening for HIV, and Prescription of Pre-Exposure Prophylaxis: Design and Implementation Study

**DOI:** 10.2196/53000

**Published:** 2024-04-15

**Authors:** Saugat Karki, Sarah Shaw, Michael Lieberman, Alejandro Pérez, Jonathan Pincus, Priya Jakhmola, Amrita Tailor, Oyinkansola Bukky Ogunrinde, Danielle Sill, Shane Morgan, Miguel Alvarez, Jonathan Todd, Dawn Smith, Ninad Mishra

**Affiliations:** 1 Division of STD Prevention Centers for Disease Control and Prevention Atlanta, GA United States; 2 Public Health Informatics Institute Decatur, GA United States; 3 OCHIN Portland, OR United States; 4 Oregon Health & Sciences University Portland, OR United States; 5 Codman Square Health Center Boston, MA United States; 6 Division of HIV Prevention Centers for Disease Control and Prevention Atlanta, GA United States

**Keywords:** clinical decision support systems, CDS, gonorrhea, pre-exposure prophylaxis, PrEP, HIV, sexually transmitted infections, electronic health records, guideline adherence

## Abstract

**Background:**

The syndemic nature of gonococcal infections and HIV provides an opportunity to develop a synergistic intervention tool that could address the need for adequate treatment for gonorrhea, screen for HIV infections, and offer pre-exposure prophylaxis (PrEP) for persons who meet the criteria. By leveraging information available on electronic health records, a clinical decision support (CDS) system tool could fulfill this need and improve adherence to Centers for Disease Control and Prevention (CDC) treatment and screening guidelines for gonorrhea, HIV, and PrEP.

**Objective:**

The goal of this study was to translate portions of CDC treatment guidelines for gonorrhea and relevant portions of HIV screening and prescribing PrEP that stem from a diagnosis of gonorrhea as an electronic health record–based CDS intervention. We also assessed whether this CDS solution worked in real-world clinic.

**Methods:**

We developed 4 tools for this CDS intervention: a form for capturing sexual history information (SmartForm), rule-based alerts (best practice advisory), an enhanced sexually transmitted infection (STI) order set (SmartSet), and a documentation template (SmartText). A mixed methods pre-post design was used to measure the *feasibility*, *use*, and *usability* of the CDS solution. The study period was 12 weeks with a baseline patient sample of 12 weeks immediately prior to the intervention period for comparison. While the entire clinic had access to the CDS solution, we focused on a subset of clinicians who frequently engage in the screening and treatment of STIs within the clinical site under the name “X-Clinic.” We measured the use of the CDS solution within the population of patients who had either a confirmed gonococcal infection or an STI-related chief complaint. We conducted 4 midpoint surveys and 3 key informant interviews to quantify perception and impact of the CDS solution and solicit suggestions for potential future enhancements. The findings from qualitative data were determined using a combination of explorative and comparative analysis. Statistical analysis was conducted to compare the differences between patient populations in the baseline and intervention periods.

**Results:**

Within the X-Clinic, the CDS alerted clinicians (as a best practice advisory) in one-tenth (348/3451, 10.08%) of clinical encounters. These 348 encounters represented 300 patients; SmartForms were opened for half of these patients (157/300, 52.33%) and was completed for most for them (147/300, 89.81%). STI test orders (SmartSet) were initiated by clinical providers in half of those patients (162/300, 54%). HIV screening was performed during about half of those patient encounters (191/348, 54.89%).

**Conclusions:**

We successfully built and implemented multiple CDC treatment and screening guidelines into a single cohesive CDS solution. The CDS solution was integrated into the clinical workflow and had a high rate of use.

## Introduction

### Background

#### Overview

Research has shown that a simultaneous, integrated approach to testing and other services targeting multiple diseases on the same population can be feasible and effective in improving accessibility and health outcomes among patients [1]. This integrated approach can be supported by point-of-care resources such as clinical decision support (CDS) system tools that can combine electronic patient health data and up-to-date guidelines and clinical protocols. This paper describes the usability phase of a study on the integration of a CDS tool for managing gonorrhea, screening for HIV, and identifying patients for HIV pre-exposure prophylaxis (PrEP) at Codman Square Clinic.

#### Gonorrhea, HIV, and PrEP

Gonorrhea was the second most commonly reported sexually transmitted infection (STI) in the United States in 2020, with a total of 677,769 cases reported to the Centers for Disease Control and Prevention (CDC) and has shown a 71.49% (395,216/677,769) increase since 2015 [2]. Among both men and women, untreated gonorrhea can cause serious and painful health problems, infertility, and in rare cases, even life-threatening conditions [3-7]. To mitigate these risks, annual screening is recommended for all sexually active women younger than 25 years and those at increased risk for infection. Timely diagnosis through routine screening and prompt, effective treatment—adhering to up-to-date CDC treatment guidelines—is paramount to both reducing gonorrhea and slowing down the threat of antimicrobial resistance [3,8-10].

HIV causes an infection that attacks the body’s immune system, specifically the cluster of differentiation antigen 4 T lymphocytes [11,12]. If left untreated, it can lead to AIDS, opportunistic infections, malignancies, and death [11]. HIV PrEP has been shown to reduce the risk of acquiring HIV infection [13,14]. As a result, the CDC has developed guidance for prescribing PrEP to individuals considered at high risk, and gonorrhea infection is identified as one of those risk factors [15,16].

#### Syndemic of STIs: Gonorrhea and HIV Infection

Patients with STIs have a 2- to 3-fold increased risk of having concomitant HIV infection compared to those without any STI [17,18]. Gonococcal infections, specifically, have also been associated with increased risk of HIV [18-23]. Gonococcal infections cause immune reactions that, among others, recruit cluster of differentiation antigen 4 cells, which can potentially enhance HIV in vivo replication and facilitate acquisition and transmission of HIV infection [20,24-26]. Previous studies have further advanced our understanding of this syndemic and describe the biological, behavioral, social, and structural determinants and their synergisms [20,22,25]. As a result, it has been a long-standing recommendation to screen patients diagnosed with STIs, including gonorrhea, for HIV [8,27].

This synergistic and potentially concomitant nature of gonorrhea and HIV and the overlap between gonorrhea treatment, HIV screening, and PrEP prescription present us with the opportunity to develop a CDC guidelines–based synergistic intervention tool that could target adequate treatment for gonococcal infections, screening for HIV based on risk factors associated with the diagnosis of gonorrhea, and PrEP prescription to relevant populations.

#### CDS Systems

CDS systems are computational tools that leverage information available in electronic health records (EHRs) to provide person-specific evidence-based information, intelligently filtered, and presented at appropriate times to help inform decisions regarding a patient’s care to improve patient outcomes and lead to higher quality care [28-31]. A systematic review of 160 articles representing 148 unique studies describing CDS implemented across diverse settings found that CDS interventions were efficacious on health care process outcomes, but data on clinical and economic outcomes were sparse [32]. The Community Preventive Services Task Force conducted a systematic review of 23 studies and reported that CDS increased HIV screening for both the general population and people at higher risk for HIV infections [33]. Based on the strong evidence of effectiveness, the Community Preventive Services Task Force has now recommended CDS to increase HIV screening [33]. The Sexually Transmitted Infections National Strategic Plan has also recommended increasing the implementation of CDS to support high-quality sexual health assessments, screen for STIs, integrated care models, and reduce adverse outcomes [34].

Gonorrhea treatment recommendations are updated regularly to represent the latest evidence to provide adequate treatment and curb the rise of antimicrobial resistance [3,8]. Keeping up to date with the latest guidelines could potentially pose a challenge to clinical providers, as evidenced with varying degrees of adherence to gonorrhea treatment guidelines [9,10]. Coupled with the synergistic nature of the infection with HIV [18,20-26], this presents an opportunity to develop a multifaceted CDS intervention to address the needs of the same patient population and improve clinical provider adherence to CDC guidelines on gonorrhea, HIV screening, and providing PrEP for HIV infection, when indicated.

### Objectives

The primary goal of this study was to translate portions of CDC treatment guidelines for gonorrhea and relevant portions of guidelines for HIV screening and PrEP prescribing, into EHR-based CDS interventions to aid clinicians’ adherence to these guidelines and improve respective patients’ health outcomes. CDC guidelines–based CDS tools for STIs have not been reported in published literature; similarly, 2 or more guidelines have not previously been cohesively built into a single CDS tool. A secondary goal was to assess whether the translated CDS solution worked in a real-world clinical workflow initiated by patients with a gonorrhea diagnosis.

## Methods

### Ethical Considerations

This project was designed and executed as a quality improvement project at OCHIN, the implementing partner, and therefore was not considered human subjects research, and institutional review board input was not obtained. The impact of the project was assessed using only aggregated deidentified data. All OCHIN members include language in their patient privacy notices stating that deidentified data may be used for research purposes. There was no compensation for human subjects’ research.

### EHR Platform and Clinical Partner

#### Overview

We partnered with OCHIN, Inc, a nonprofit community-based health center–controlled network as the EHR provider [35,36]. We selected OCHIN’s Epic EHR [37] as it serves as the primary EHR to almost 1000 health care sites with 21,000 clinicians in 45 states who serve over 6 million patients [38]. OCHIN provides 1 instance of Epic consisting of enterprise-wide master patient index; patients have a single medical record across all clinics in the network, and all data are managed centrally.

Within the OCHIN network, we partnered with Codman Square Health Center, a Federally Qualified Health Center in Dorchester, Massachusetts, to pilot the CDS intervention [39]. We selected Codman Square Health Center as both patients with gonorrhea and HIV are regularly managed in their clinics and they have a well-established STI-reporting practice in place. Typically, they have over 115,000 client contacts per year.

#### Intervention Clinic

While all of Codman Square Health Center had access to the CDS solution, a subset of clinicians within the Internal Medicine clinic, specifically, were selected as the focus of the intervention. This subset of clinicians frequently engaged in the screening and treatment of STIs and provide care under the name “X-Clinic” to allow a level of discretion to the patients they serve. The X-Clinic clinicians were selected for the focus of the intervention due to the high rates of gonorrhea infection and the potential for use of the CDS solution during the pilot period. A clinical champion was identified to provide insight and encourage participation among staff along with 2 project leads, 1 representing the providers and 1 for support staff. While the CDS tool was available to the broader Codman Square Health Center, the X-clinic received targeted training and support to use the tool. This training and support were not extended to the rest of Codman Square Health Center.

### CDS Solution Design

#### Study Design

An internal review by OCHIN, the implementing partner, determined this work as quality improvement and deemed that an institutional review board review was not necessary. A 12-week study period was planned, beginning August 31, 2021, and ending on November 23, 2021. A mixed methods pre-post design [40] was used to measure the *feasibility*, *use*, and *usability* of the CDS solution. During the time of the planned intervention, COVID-19 altered the typical pattern of patients seeking STI-related care [41,42]. As a result, we determined that the 12-week period immediately prior to the intervention period would be the most representative sample of baseline patient data.

#### Practice Coach Engagement

In addition to the direct training provided, the X-Clinic clinicians received targeted and continual support on the use of the CDS solution using the practice coaching methodology. This included supporting adaptive skills and learning to build capacity and capability for the care team to effectively use the CDS solution, explore change ideas, provide feedback to support the team’s progress as the team becomes more self-sufficient, and engage with iterative learning. Practice coaching (also known as practice facilitation) has been demonstrated to be effective in successfully implementing tools and new evidence into clinical practice [43-46]. A key part of the intervention design included a dedicated practice coach who regularly engaged with the clinical champion and project leads at the X-Clinic. The coach was part of the project team’s kick-off meeting, which included a warm welcome from the CDS solution developer, project orientation from the project manager, training by the trainer to demonstrate the solution, and an overview of the coaching engagement from the coach. The facilitation meetings were held biweekly for the duration of the project, and they typically included discussions about general assessment and feedback, use, perceived effectiveness, and clinical integration of the CDS solution. When available, CDS solution–related data were reviewed for discussion of key insights, opportunities for improvement, and when a need was identified, the practice coach engaged subject matter experts to provide added support and facilitated referrals for technical and application support.

#### CDS Solution Development

OCHIN Epic EHR [37] has native tools to develop CDS solutions in a variety of formats, each performing specific functions. For this CDS solution, we developed four tools: (1) a sexual history data capture form using “SmartForm” (Epic’s name for a method for capturing responses to questions in a structured format) and a patient questionnaire with the same questions that can be made available to the patient prior to the visit, (2) a “best practice advisory” (BPA; Epic’s name for rule-based alerts), (3) an enhanced STI order set—that included diagnoses as well as links to documentation templates—using “SmartSet” (Epic’s name for order sets), and (4) a documentation template using “SmartText” (Epic’s name for a method of notetaking that prompts clinician with standard information collected and presented during a visit).

**Figure 1:**
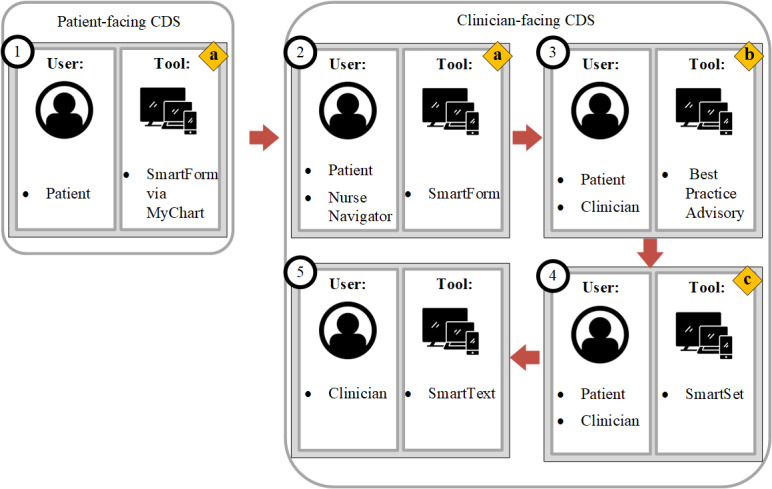
Workflow and details of the clinical decision support solution. CDS solution workflow; represented in numerical order in the diagram: 1. "SmartForm" (a native Epic tool for capturing responses to questions) captured detailed sexual history from the patient. It was made available to patients in advance via MyChart (Epic’s patient portal) on their phone app or web browser. 2. At the beginning of the visit, the nurse navigator entered a reason for the visit and chief complaint, reviewed information entered via SmartForm, and completed the SmartForm, if incomplete. 3. When the clinical provider accessed the patient’s chart, a "best practice advisory" (BPA; Epic’s name for rule-based alerts) directed the clinical provider to a "SmartSet" (Epic’s name for order sets) that represented the Centers for Disease Control and Prevention treatment and prevention guidelines for gonococcal and HIV infections. 4. If the clinician opted to open the SmartSet, a detailed list of orders, treatment, and diagnosis options was provided. 5. The clinician documented the visit using a "SmartText" (Epic’s name for a method of taking clinical notes) documentation template specifically designed for sexually transmitted infections (STIs) to standardize the information collected and presented during the visit. Gonorrhea treatment, HIV screening, and PrEP (pre-exposure prophylaxis) prescription guidelines, represented in alphabetical order in the diagram: a. If the patient was considered at risk for HIV infection, additional questions related to PrEP prescriptions appeared in the SmartForm. b. The BPA was displayed to the clinician if the patient met the criteria to treat for gonococcal infection. c. The clinical provider was offered to use the SmartSet which included guidance for screening and treating STIs, and screening and monitoring for PrEP. CDS: clinical decision support.

We developed a CDS solution by integrating translated information from 3 separate CDC guidelines, specifically to guide clinicians to choose appropriate therapy for gonorrhea [3,8] and—as per the CDC’s guidelines to prevent or diagnose new HIV infections [47-49] and link those individuals considered at risk to relevant prevention and medical services [15,16]—prompt them to screen for HIV and consider PrEP, if patients met those criteria (Figure 1). Health data standards were used wherever possible; *ICD-10-CM* (*International Classification of Diseases, Tenth Revision, Clinical Modification*) was used to evaluate the diagnosis of gonococcal infections.

The Sexual History SmartForm and corresponding patient questionnaire were developed to collect sexual history and STI-related symptoms from patients. The form and questionnaire were structured as point and click questions and answers for ease of use; patients had access to the questionnaire via MyChart [50] (Epic’s patient portal) prior to the visit (Figure 1, step 1; Table 1). At the beginning of the clinic visit, nurse navigators reviewed the information collected using the SmartForm and completed the questionnaire, if incomplete (Figure 1, step 2). Based on CDC’s guidelines [15,16,48], patients were considered to be at risk for HIV infection for the following criteria: (1) positive result or diagnosis for gonococcal infection in the last 6 months; (2) reason for visiting the clinic was related to STI; and (3) sexual risk behaviors, known partner with any STI, and if patient or partner using or sharing needles. If the patient’s response suggested a high risk for HIV infection, the SmartForm algorithm presented further questions to gauge awareness and interest for PrEP (Table 1).

**Table 1:** Information collected, criteria, and type of decision support provided by each clinical decision support tool.

Clinical decision support tool	Type of information collected	Criteria for providing clinical decision support	Clinical decision support provided
SmartText	Prefilled template to document clinical encounter for STI^a^-related visit	SmartSet recommendation to use SmartText	SmartForm was embedded in SmartText
SmartForm	Sexual history STI symptoms	Positive laboratory result for gonococcal infection in the last 6 months Diagnosed with gonococcal infection in the last 6 months Reason for visiting the clinic was related to STIs Current partner has HIV or AIDS Patient or partner using or sharing needles in the last 6 months Syphilis or gonorrhea in the last 6 months Infrequent use of condoms with partners at risk of HIV (persons who inject drugs or men who have sex with men), Male who has sex with males who has had anal sex without condoms in the last 6 months (outside of a monogamous relationship with an HIV-negative partner) Male who has sex with males who has had any STI (including chlamydia)	Additional PrEP^b^-related questions prompted: “Have you heard of HIV PrEP?” “Have you ever taken HIV PrEP?” “Are you interested in HIV PrEP?”
Best practice advisory	SmartForm: Sexual history STI symptoms EHR^c^: Prior testing history Chief complaint Treatment information	Confirmed positive result for gonococcal infection in the last 14 days Chief complaint related to gonorrhea STI symptoms or concern Penile, urethral, rectal, or vaginal discharge STI and HIV-related screening, testing, follow-up, or visit Treated presumptively due to a partner with known gonococcal infection	Alerted clinical provider to use SmartSet to aid clinical decisions
SmartSet	SmartForm: Sexual history STI symptoms EHR: Prior testing history Chief complaint Treatment information	Best practice advisory recommendation to use SmartSet	Recommended laboratory tests to screen for STI and HIV Recommend PrEP to at-risk patients Provide recommendations for medication regimens to treat gonococcal infections Provide a link to SmartText

^a^STI: sexually transmitted infection.

^b^PrEP: pre-exposure prophylaxis.

^c^EHR: electronic health record.

For patients who (1) had a confirmed positive result for gonococcal infection in the last 14 days, (2) had a chief complaint related to gonorrhea (STI symptoms or concern; penile, urethral, rectal, or vaginal discharge and STI-related screening, testing, follow-up, or visit), or (3) were treated presumptively due to a partner with known gonococcal infection, a BPA would alert the clinical provider (Figure 1, step 3) to use the SmartSet to assist in providing standardized clinical care (Figure 1, step 4; Table 1). The SmartSet was designed to (1) recommend laboratory tests to screen for STI, including HIV, (2) provide PrEP prescription options, (3) present CDC guidelines–based recommendations for medication regimens as preferred options to treat gonococcal infections, and (4) provide a link to gonorrhea treatment guidelines for anything beyond uncomplicated gonorrhea and a link to the SmartText (Figure 1, step 5; Table 1). Opening the BPA did not necessarily mean that clinical providers selected diagnoses or ordered laboratory tests or medication directly from the SmartSet but merely that those options were made available for selection and action. Diagnoses, laboratory tests, and medication orders could also be entered for the patient outside of the SmartSet. The SmartText template was developed to prompt the clinical provider to document in a standardized manner (Table 1). The SmartText included common heading for note taking, prepopulated STI-related laboratory results from the patient’s chart, and risk reduction suggestions.

### Use of CDS Solution and Outcomes of Intervention

We evaluated CDS solution *use* and outcome metrics specifically for any person with confirmed case of gonorrhea or with an STI-related chief complaint. Data were collected on the demographics of the patient population to ensure appropriate comparison between the baseline and intervention populations, *use* metrics of CDS solutions by X-Clinic, and outcomes following the implementation of the CDS solution. To measure patient and disease characteristics for the baseline period (immediate 12-week period prior to the intervention period), a 1-time data extraction was performed (Table 2). During the intervention period, quantitative data were extracted on a nightly basis.

**Table 2:** Goal of assessing the clinical decision support solution and their respective data sources.

Assessment area and data source		Assessment question
**Demographics**		
	Baseline period: 1-time data extraction	What are demographic characteristics of the patient population in the baseline period?
	Intervention period: nightly data extraction	What are demographic characteristics of the patient population in the intervention period and how do they compare with the patient population in the baseline period?
**Use**		
	Intervention period: nightly data extraction	Are clinical providers using the CDS^a^ solution and how are they using it?
	Midpoint survey: questionnaire for X-Clinic clinical providers	Identify challenges for course correction, if necessary
**Outcomes**		
	Intervention period: nightly data extraction	Are there any changes in outcomes of gonorrhea treatment, screening for HIV and prescription of HIV PrEP^b^?
**Usability**		
	Midpoint survey: questionnaire for X-Clinic clinical providers	What are the provider perspectives on the characteristics of the CDS?
	End of intervention period: key informant interviews	Which features do the clinicians like or do not like? How did these factors influence use?

^a^CDS: clinical decision support.

^b^PrEP: pre-exposure prophylaxis.

### Clinical Provider Perspectives and Usability of the CDS Solution

We conducted 1 midpoint survey to confirm the *use* of CDS solution and identify any challenges faced by the clinical providers using the solution (Table 2; Multimedia Appendix 1). Four end users—or clinical providers who used the CDS solution—completed the survey. We also conducted key informant interviews with 3 end users at the end of the pilot period to assess the *usability* of the CDS solution (Multimedia Appendix 2). The CDS solution design encompassed multiple end user roles such as the SmartForm, which was anticipated to be completed by nurse navigators, and SmartSet, which was anticipated to be completed by clinical providers. We interviewed individuals who represented the various roles such as leadership, clinical provider, and nurse navigator perspectives. The purpose of the key informant interviews was to understand treatment and screening workflows prior to CDS solution implantation, perception, and impact of the CDS solution and solicit suggestions for potential future enhancements (Table 2). We measured the *use* and *usability* of the CDS solution based on the clinical provider role to reveal how perception and familiarity with the CDS solution may have impacted use.

### Data Analysis

We used quantitative and qualitative means to examine the *feasibility* of this guidelines-based CDS solution in a real-world clinical workflow, where 2 separate guidelines have been cohesively built into a single CDS tool solution, initiated by patients with a gonorrhea diagnosis. Quantitative data were used to describe the *use* metrics and outcomes post implementation of CDS solution and were summarized by descriptive statistics using counts and percentages. To assess *usability* of the CDS tool, qualitative data were systematically coded from interview transcripts, and findings were determined using a combination of explorative and comparative analysis to examine end user (clinical provider) perspectives.

Statistical analysis was conducted to compare the differences between patient populations in the baseline and intervention periods. For continuous variables, to test for a statistically significant difference among populations, a 2-sample *t* test was used. To test for a statistically significant association among outcomes, all categorical variables were tested using Pearson chi-square test, and if an expected count of 5 was not observed for a cell, Fisher exact test was used. If a statistically significant association was observed, odds ratios were calculated. All statistical analyses were conducted using SAS (version 9.4; SAS Institute).

## Results

### CDS Solution Design

#### Description of Patient Populations: Pre- and Post-CDS Intervention

During the intervention period, 12,048 patients were provided with clinical care in Codman Square Health Center and 37 were diagnosed with gonorrhea; similarly, 40 patients out of 11,269 were diagnosed with gonorrhea in the baseline period. The mean age of patients seen in the baseline and intervention periods was 37 (SD 21.46) years and 36 (SD 21.98) years, respectively (*P*<.001). A 2-sample *t* test analysis of the age groups revealed that the intervention period included younger individuals compared to the baseline period and cannot be considered similar; however, the largest age group of 25-44 years old remained the same with 30.78% (3469/11,269) in the baseline period and 29.58% (3564/12,048) in the intervention period. Using Fisher exact test, it was determined that the patients in the baseline and intervention periods were comparable regarding gender (*P*=.25). While ethnicity distribution was found to be similar (*P*=.56) using Pearson chi-square test, baseline and intervention groups differed significantly by race (*χ*_1_^2^=4.36; *P*=.04). Nevertheless, the largest patient population served by Codman Square Health Center was Black or African American in both baseline (9040/11,269, 80.22%) and intervention (9565/12,048, 79.39%) periods.

### Use of CDS Solution and Outcomes of Intervention

#### Use

**Figure 2:**
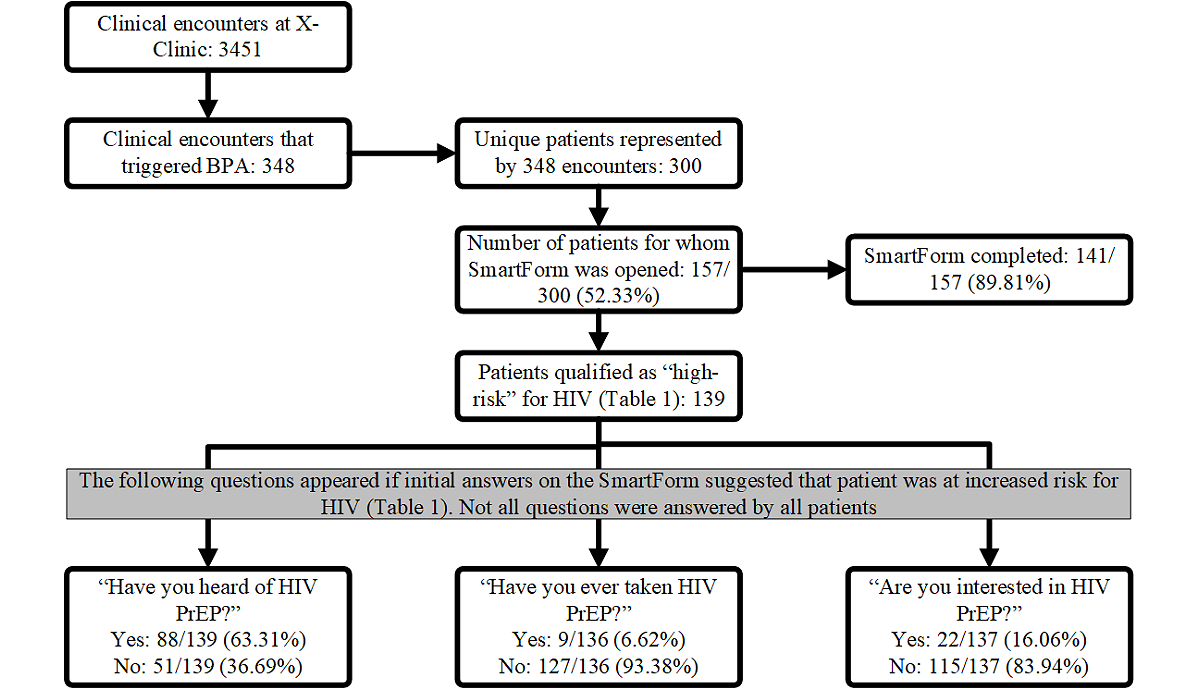
Description of patient interaction with SmartForm, percent completed, and answers selected. BPA: best practice advisory; PrEP: pre-exposure prophylaxis.

Throughout Codman Square Health Center, the BPA was triggered 950 (4.07%) times. Within the X-Clinic specifically, the BPA was triggered in one-tenth of all the clinical encounters (348/3451, 10.08%); these 348 encounters represented 300 patients (Figure 2). The SmartForm was opened for about half of the patients (157/300, 52.33%), and in instances when the SmartForm was opened, it was completed 89.81% (141/157) of the time (Figure 2). Of note, while the BPA did not prompt the user to open the SmartForm, the patient cohort where the BPA presented was used for analysis as this represents the target population for this study. For those patients whose responses determined them as high-risk for HIV, further PrEP-related questions were asked (Table 1). Some information about PrEP was known to 63.31% (88/139) of the patients who were asked that question; 6.62% (9/136) of patients reported having taken PrEP and 16.06% (22/137) patients showed interest in PrEP (Figure 2).

Clinical providers could open SmartSet from the BPA (Figure 1, step 4), and this action was taken for about half of the patients (162/300, 54%) for whom BPA was presented. In the target population, the most common diagnosis was “Vulvovaginitis” (*ICD-10-CM*: N76.0); it was assigned to 34 patients, 21 were assigned to patients for whom BPA was presented, and 13 were assigned where no BPA was presented (Table 3). Diagnoses were assigned, and laboratories were ordered both from BPA-prompted SmartSet and outside of BPA without using the CDS solution (Table 3). No patients were diagnosed with cervicitis but were diagnosed with urethritis, pharyngitis, and proctitis. The most ordered laboratory test was for HIV. More than half (191/348, 54.89%) of the encounters where a BPA was presented received these orders for HIV testing.

In the X-Clinic, SmartText note template was used in most of the clinical encounters (313/348, 89.9%), where the BPA presented. Clinical providers used SmartText extensively (2343/3103, 75.5%) even during encounters where the BPA did not trigger. The SmartText was accessible either through the SmartSet or a standard “quick button” in the documentation area. The clinical site continued to use the CDS tool beyond the study period.

**Table 3:** Use of best practice advisory by diagnosis in the X-Clinic.

Diagnoses selected and tests ordered from SmartSet			Best practice advisory presented (n=302), n (%)		Best practice advisory not presented (n=3103), n (%)		Total (n=3451), n (%)
**Urethritis**							
	Urethritis, unspecified [N34.2]	5 (62.5)		3 (37.5)		8 (0.23)	
	Gonococcal urethritis [A54.01]	0 (0)		0 (0)		0 (0)	
	Chlamydial urethritis [A56.01]	0 (0)		1 (100)		1 (0.03)	
**Cervicitis**							
	Cervicitis [N72]	0 (0)		0 (0)		0 (0)	
	Gonococcal cervicitis [A54.03]	0 (0)		0 (0)		0 (0)	
	Chlamydial cervicitis [A56.09]	0 (0)		0 (0)		0 (0)	
**Vulvovaginitis**							
	Vulvovaginitis [N76.0]	21 (61.76)		13 (38.24)		34 (0.99)	
	Gonococcal vulvovaginitis, unspecified [A54.02]	0 (0)		0 (0)		0 (0)	
	Chlamydial vulvovaginitis [A56.02]	0 (0)		0 (0)		0 (0)	
**Pharyngitis**							
	Pharyngitis, unspecified etiology [J02.9]	4 (28.57)		10 (71.43)		14 (0.41)	
	Pharyngitis, gonococcal [A54.5]	3 (75)		1 (25)		4 (0.12)	
	Pharyngitis, chlamydial [A56.4]	0 (0)		0 (0)		0 (0)	
**Proctitis**							
	Proctitis [K62.89]	1 (50)		1 (50)		2 (0.06)	
	Proctitis, chlamydial [A56.3]	1 (50)		1 (50)		2 (0.06)	
	Proctitis, gonococcal [A54.6]	1 (100)		0 (0)		1 (0.03)	
**Sexually transmitted infection–related laboratory test**							
	Chlamydia or Gonorrhoeae RNA, TMA^a^, and throat	36 (72)		14 (28)		50 (1.45)	
	Chlamydia or Gonorrhea, RNA, TMA and rectal	19 (55.88)		15 (44.12)		34 (0.99)	
	DNA Probe, chlamydia or gonorrhea, swab	1 (50)		1 (50)		2 (0.06)	
	Chlamydia/Gonorrhea BVS^b^ (BHN COD)	4 (80)		1 (20)		5 (0.14)	
	Chlamydia/Gonorrhea, DNA, SDA^c^, urine	12 (32.43)		25 (67.57)		37 (1.07)	
	Syphilis IgG^d^/IgM^e^ screen w/reflex to RPR^f^	30 (29.7)		71 (70.3)		101 (2.93)	
	HIV fourth-generation ELISA^g^	191 (38.74)		302 (61.26)		493 (14.29)	

^a^TMA: transcription-mediated amplification.

^b^BVS: blind vaginal swab.

^c^SDA: strand displacement amplification.

^d^IgG: immunoglobulin G.

^e^IgM: immunoglobulin M.

^f^RPR: rapid plasma reagin.

^g^ELISA: enzyme-linked immunosorbent assay.

#### Outcomes of Intervention

In the intervention period, among patients where a BPA was triggered, there were fewer patients with a diagnosis of gonococcal infections (33/750, 4.4%) as compared to the baseline period (41/649, 6.32%; Table 4). While roughly four-fifths (34/41, 82.93%) of these patients diagnosed with gonorrhea were prescribed antimicrobials for treating the infection, the difference in the number of patients treated in the intervention (26/33, 78.79%) and baseline periods were not statistically significant (*P=*.65). Individuals in the intervention period had a 26% decrease in odds (odds ratio 0.74, CI: 0.60-0.91) of being screened for HIV (310/750, 41.33%) compared to the baseline period (317/649, 48.84%). PrEP was prescribed 2 times more to patients in the intervention period (12/750, 1.6%) compared to the baseline period (6/649, 0.92%); however, analysis revealed that these numbers not to be statistically significant (*P*=.26).

**Table 4:** Outcomes of intervention, pre- and postclinical decision support solution intervention.

Characteristic			Baseline period (n=11,269), n (%)		Intervention period (n=12,048), n (%)
Target population^a^			649 (5.76)		750 (6.23)
**Diagnosed with gonorrhea**			41 (6.32)		33 (4.4)
	Prescribed with antimicrobial treatment	34 (82.93)		26 (78.49)	
**Screened for HIV**			317 (48.84)		310 (41.33)
	Diagnosed with HIV	3 (0.95)		3 (0.97)	
**Prescribed PrEP^b^**			6 (0.92)		12 (1.6)
	Truvada	5 (83.33)		11 (91.67)	
	Descovy	1 (16.67)		1 (8.33)	

^a^Any person with confirmed case of gonorrhea or with a sexually transmitted infection–related chief complaint.

^b^PrEP: pre-exposure prophylaxis.

### Clinical Provider Perspectives and Usability of the CDS Solution

#### Midpoint Survey

Most of the survey responders reported that the CDS solution was not intrusive to their clinical practice workflow (2/4, 50% strongly agreed; 1/4, 25% agreed; and 1/4, 25% was neutral). They also agreed (0/4, 0% strongly agreed; 3/4, 75% agreed; and 1/4, 25% was neutral) that the CDS solution was easy to navigate and provided sufficient time to use it during the patient consultation. They had neutral responses (0/4, 0% strongly agreed; 1/4, 25% agreed; and 3/4, 75% was neutral) about the CDS solution’s ability to present clear recommendations for treatment.

#### Key Informant Interviews

The interview revealed 3 major emerging themes. First, the interviewees opined that the CDS solution provided a faster and more standardized approach for capturing the sexual history and treating the patient and planned to keep using it after the pilot was complete. Second, while the CDS solution improved the efficiency and streamlined their existing processes, the interviewees did not change their treatment or screening recommendations based on the solution. Third, they also revealed that the full scope of the CDS solution was not clear to them and reported discovering components of the solution on their own that they were not aware were part of the CDS solution.

There were 3 key themes related to the features of the CDS solution. First, the BPAs alone would not prompt behavior change and use. Interviewees emphasized the importance of education of new guidelines and changes in the EHR tools from someone they know and trust and believed this would promote adherence from clinicians. Second, there were mixed reviews regarding the use of the logic that determined to present PrEP-related questions to some patients considered at high-risk for HIV. While 1 interviewee noted that the unique focus of X-Clinic to specifically address sexual health–related questions and conditions, all patients should be asked PrEP-related questions and noted confusion over the questions being presented to only some patients and not to other patients. Another interviewee indicated that this logic was helpful for colleagues who are less familiar with PrEP to highlight relevance in populations not typically targeted for PrEP, such as heterosexual patients. Third, there was a general sense that SmartSets were hard to navigate. Multiple interviewees commented that they were unable to find SmartSets without BPAs, and once in the SmartSet, it was not easy to leave and come back if an issue outside the scope of the SmartSet needed to be addressed, such as discussions regarding intrauterine devices in relation to gonococcal infections. One interviewee noted handwriting details to remember after completing a task within SmartSet.

#### Role-Based Use of CDS Solution

A patient may be seen by multiple providers from more than one provider type category. In the target population (750/12,048, 6.23%) described in Table 4, 32 Doctors of Medicine and Doctors of Osteopathy (MD/DO) provided care for 281 patients and minimally used SmartForms (20/281, 7.12%) but completed most of them once opened (18/20, 90%); 26 advanced practice providers (APPs) provided care to 362 patients, used SmartForms for 20.99% (76/362) of patients, and 81.58% (62/76) completed most of them. In contrast, the “other” category, which included all other clinical staff such as case manager, dentist, laboratory technician, licensed practical nurse, medical assistants, midwives, and social workers, had the highest use, opening SmartForms for half of the patients they served (99/192, 51.56%) and completed most of the forms (83/99, 83.84%). Similarly, “other” category staff administered the highest number of PrEP-related questions, followed by APPs and MD/DOs administered very few. Providers classified as MD/DOs had 309 clinical encounters with 281 patients and opened SmartSets in half of the encounters (157/309, 50.81%) and those classified as APPs had 398 clinical encounters with 362 patients and opened SmartSets slightly lower than half of the encounters (180/398, 45.23%). “Other” staff opened SmartSets the least (82/126, 39.42%), with 10 staff who interacted with 192 patients in 208 encounters.

## Discussion

### Principal Findings

We integrated translated information from 3 CDC guidelines into a single cohesive CDS solution. This included CDC’s treatment recommendations for gonococcal infections (for appropriate diagnosis, testing, and treatment for gonorrhea) and HIV screening recommendations and PrEP prescription recommendations that stem from the diagnosis of gonorrhea. This CDS solution was implemented at a federally qualified health center where it continued to be used after the intervention period. The CDS solution successfully collected relevant information about the patient, evaluated the patient information against the CDC guidelines to prompt adequate treatment for gonorrhea, and identified at-risk patients for further HIV screening and PrEP prescription. The quantitative data revealed high rates of *use* of the various tools developed for the CDS solution and demonstrated the *feasibility* of incorporating such multifaceted guidelines–based CDS solutions in real-world clinical settings.

During our *usability* testing, clinical providers used all components of the CDS solution, with varying degrees, based on their respective provider type or roles, and familiarity with the CDS solution. Providers classified as MD/DO and APP used the SmartSet more than “other” staff such as medical assistants and social workers and vice versa for the SmartForm that performed the initial screen for patients’ sexual history and PrEP awareness. In the X-Clinic, specifically, the BPA was presented in 10.08% (348/3451) of the encounters; clinicians reported that this was not intrusive and could be used as a benchmark to potentially reduce alert fatigue. The SmartSet was opened in about half of the times alerted by BPA, but fewer orders were directly entered from the SmartSet itself. This does not mean that patients did not receive care. Some insights offered by providers as plausible explanations include (1) clinicians reported benefiting from the reminder and served as a knowledge source even if they performed those actions outside of the SmartSet; (2) clinicians were not accustomed to using SmartSets and reported experiencing difficulties in locating it once they moved away from it during the clinical visit; (3) the BPA alert at the time when the patient record was initially opened may be too early to determine diagnosis, order laboratory tests, and prescribe treatment, delaying the timing of the BPA could be explored; and (4) clinicians had neutral responses when asked about CDS solution’s ability to present clear recommendations since SmartSet presented all treatment options with those aligned to the guidelines marked as “preferred” instead of targeted recommendations for a particular patient.

There was no significant difference between the baseline and intervention patient groups with respect to the treatment for gonococcal infections. It is important to note that X-Clinic is primarily focused on providing sexual health and STI-related care, and the providers were well versed with treatment recommendations, which could be an explanation for the lack of difference. The results also show that one-fifth of the patients in both baseline and intervention periods were not treated with antimicrobials during the visit. A likely explanation is that these patients were empirically treated elsewhere and were referred to X-Clinic for further follow-up, given their specialty in providing STI-related care. Fewer patients were screened for HIV during the intervention period compared to the baseline period. However, key informant interview participants anecdotally reported an increased interest regarding PrEP in less traditional populations, such as heterosexual women. While PrEP was prescribed to twice as many patients in the intervention period compared to the baseline period, this was not deemed statistically significant.

The widespread adoption of EHRs has provided us with the unique opportunity to leverage CDS approaches to automate and align clinical decisions such as identifying at-risk patients for further screening and providing adequate treatment with the latest guidelines. This becomes especially important in situations where clinical providers may not be familiar with the latest screening and treatment guidelines. Given the rise in resistance to antimicrobial treatment for gonorrhea, adherence to treatment guidelines is even more important. Further development of standards and tools and examination of workflows to implement useful guidelines-based CDS solutions with the ultimate goal of improving patient care are needed.

### Limitations

The original design was based upon a 6-month intervention period. A longer pilot period would have allowed the opportunity for a larger data set of patients diagnosed with gonorrhea and potentially more compelling statistical correlations, along with additional time for the clinical staff to acclimate to the new solution.

For this phase of our work, it was important to test the *feasibility* of the guidelines-based CDS logic, measure the use of the CDS tools, and solicit feedback from end users regarding their experience using the CDS tool. Prioritizing these factors, we decided to leverage EHR-native but vendor-specific tools such as SmartForm, BPA, SmartSet, and SmartText. We factored that OCHIN, our partner organization has many clinics using their instance of Epic EHR across the United States [37,38], and any lessons learned could potentially be replicated and scaled to other interested clinical sites.

Since the focus of this work was to translate multiple CDC guidelines into a cohesive CDS solution, we selected a clinical site that was well versed in STI diagnosis and treatment with a track record of serving a large number of patients seeking sexual health and STI-related care. Due to the same reasons, this clinical setting may not be the best target of clinical providers to measure any potential outcomes of the CDS solution. The CDS solution would better serve clinical providers and settings, where there is less familiarity of treating patients with STIs.

In this study, we report the *feasibility*, *use*, and *usability* of the CDS solution; we did not measure the effectiveness of the intervention. We did not examine any change in clinical practice and its impact on clinical care or the long-term prevention outcomes in the patient population. As a result, we did not randomize the study and did not examine any potential confounding. Similarly, while we obtained quantitative and qualitative information from end users of various roles, the small sample size poses challenges to draw firm conclusion of usability at scale. Further examination of the CDS solution could be performed by manual chart review with an annotated gold standard measure.

### Comparison With Prior Work

In the past, CDC guidelines–based CDS interventions have been designed to recommend appropriate immunizations [51], screening for alcohol use disorder [52], and screening for cervical cancer [53]. A United States Indian Health Services clinic implemented a chlamydia and HIV screening tool and observed increase in screening frequencies of both chlamydia and HIV; however, this was not strictly based on CDC guidelines [54]. Another study described the implementation of CDS tool to encourage appropriate prescription of azithromycin in primary care clinics with the aim to curb rise of antimicrobial resistance, but this study was not targeted toward STIs and was focused on bronchitis and upper respiratory tract infections [55]. To the best of our knowledge, no CDS tools have been implemented with the goal of improving adherence to guidelines for gonorrhea treatment, HIV screening, and HIV PrEP.

### Conclusions

It is feasible to integrate multiple CDC guidelines into a single cohesive CDS solution. The CDS solution showed high rates of use, but given the short study period, we could not adequately measure realistic patient outcomes. The clinical site has opted to continue using the full scope of the CDS solution, and perhaps that decision is a measure of success.

Learning from this experience, we will be developing a standards-based EHR-agnostic CDS solution with a longer study period.

## Appendix

Multimedia Appendix 1 Midpoint survey instrument.

Multimedia Appendix 2 Key informant interview questions.

## Abbreviations

APP: advanced practice provider
BPA: best practice advisory
CDC: Centers for Disease Control and Prevention
CDS: clinical decision support
DO: Doctor of Osteopathy
EHR: electronic health record
ICD-10-CM: International Classification of Diseases, Tenth Revision, Clinical Modification
MD: Doctor of Medicine
PrEP: pre-exposure prophylaxis
STI: sexually transmitted infection

## Data Availability

All data generated or analyzed during this study are included in this published article and its supplementary information files.
