# Supplementary material for: Clinical Decision Support System for Guidelines-Based Treatment of Gonococcal Infections, Screening for HIV, and Prescription of Pre-Exposure Prophylaxis: Design and Implementation Study
Source: JMIR Form Res. 2024 Apr 15;8:e53000. doi: 10.2196/53000 (PMC11058559; doi:10.2196/53000)
Supplement: Multimedia Appendix 1 [file formative_v8i1e53000_app1.docx]

# Appendix 1: Midpoint survey instrument

Survey questions:

The follow questions used a rating scale of Strongly Agree, Agree, Neutral, Disagree and Strongly Disagree.

- Using an GC/HIV/PrEP screening clinical decision support tool disturbs the patient
- I believe using an GC/HIV/PrEP screening clinical decision support tool is not an intrusion to clinical practice
- I do not need a GC/HIV/PrEP screening clinical decision support tool because I can rely on my personal expertise
- I do not need a GC/HIV/PrEP screening decision support tool because it does not work well with complexities in patients’ care
- I received training on the GC/HIV/PrEP screening clinic decision support tool that was tailored to my needs
- I have sufficient time to use the GC/HIV/PrEP screening clinic decision support tool during the patient consultation
- I believe the GC/HIV/PrEP screening recommendations considers the holistic aspect of the patient
- The GC/HIV/PrEP screening clinical decision support system provides me an explanation for the GC/HIV/PrEP CDS testing recommendation as relevant to my patient
- The GC/HIV/PrEP screening clinical decision support tool is visually easy to navigate
- I receive timely alerts from my GC/HIV/PrEP screening clinic decision support tool during the patient visit
- The GC/HIV/PrEP screening clinical decision support tool is intuitive to use
- I believe using a GC/HIV/PrEP screening clinical decision support tool helps make the clinical encounter with the patient more efficient
- The GC/HIV/PrEP screening clinical decision support tool is well-integrated in my clinical workflow

The following questions were open ended.

- Please provide feedback or comment about the Best Practice Advisory (BPA) alert
- Please provide feedback or comment about the SmartSet
- Please provide feedback or comment about the SmartForm
- Please provide any other general feedback or comment on the tool
- Name of Respondent
