# Supplementary material for: Clinical Decision Support System for Guidelines-Based Treatment of Gonococcal Infections, Screening for HIV, and Prescription of Pre-Exposure Prophylaxis: Design and Implementation Study
Source: JMIR Form Res. 2024 Apr 15;8:e53000. doi: 10.2196/53000 (PMC11058559; doi:10.2196/53000)
Supplement: Multimedia Appendix 2 [file formative_v8i1e53000_app2.docx]

# Appendix 2: Key informant interview questions

Sexually Transmitted Infection Clinical Decision Support (CDS) Module Clinic Champion Key Informant Interview (KII) Questions

1. Please state your name and job title.
2. What is your primary role within the organization?
   1. How long have you been in your current role within the organization? If you have multiple roles, please answer based on your primary role.
3. How often do you login into the OCHIN Epic EHR system? (daily, weekly, etc.)
4. In your current role at this facility, approximately how many hours a week do you spend providing direct patient care (i.e., activities related to the prevention, diagnosis, or treatment of disease) specific to gonorrhea treatment, HIV screening or PrEP prescriptions?
5. Did you/your team use the various CDS components when treating a patient with symptoms of Gonorrhea?
6. Can you describe how the CDS influenced your practices when serving patients Gonorrhea, HIV screening, PrEP? Please explain why or why not?
   1. How did you change your treatment of gonorrhea based on the new CDS solution?
   2. Did the CDS solution clearly encourage HIV screening after a gonorrhea diagnosis? Please explain why or why not.
   3. Did the CDS solution help to identify new patients who are eligible for PrEP? Please explain why or why not.
7. How well does the CDS fit within your existing clinical workflow?”
   1. What were the benefits?
   2. How complicated was it to use the CDS?
   3. What kinds of changes or alterations would you suggest to be made to the CDS to meet the needs of your patient population?
8. During the pilot implementation, what challenges did you/your team encounter?
   1. How did you resolve the challenges?
   2. If challenges exist, have the steps for resolving these challenges been identified?
      1. If yes, describe those steps.
9. What is the most important thing you would tell someone, or would want someone to know, before adopting the clinical decision support solutions for STI?
10. Are there any final lessons or best practices learned that you would like to share?

Is there anything else about CDS that we haven’t asked about that you would like to share?
